# Neutrophil roles in left ventricular remodeling following myocardial infarction

**DOI:** 10.1186/1755-1536-6-11

**Published:** 2013-06-03

**Authors:** Yonggang Ma, Andriy Yabluchanskiy, Merry L Lindsey

**Affiliations:** 1 San Antonio Cardiovascular Proteomics Center, San Antonio, TX, USA; 2 Jackson Center for Heart Research, Department of Physiology and Biophysics, University of Mississippi Medical Center, Jackson, MS, USA; 3 Research and Medicine Services, G.V. (Sonny) Montgomery Veterans Affairs Medical Center, Jackson, MS, USA

**Keywords:** PMNs, Myocardial infarction, Inflammation, Innate immunity, Degranulation, Matrix metalloproteinases

## Abstract

Polymorphonuclear granulocytes (PMNs; neutrophils) serve as key effector cells in the innate immune system and provide the first line of defense against invading microorganisms. In addition to producing inflammatory cytokines and chemokines and undergoing a respiratory burst that stimulates the release of reactive oxygen species, PMNs also degranulate to release components that kill pathogens. Recently, neutrophil extracellular traps have been shown to be an alternative way to trap microorganisms and contain infection. PMN-derived granule components are also involved in multiple non-infectious inflammatory processes, including the response to myocardial infarction (MI). In this review, we will discuss the biological characteristics, recruitment, activation, and removal of PMNs, as well as the roles of PMN-derived granule proteins in inflammation and innate immunity, focusing on the MI setting when applicable. We also discuss future perspectives that will direct research in PMN biology.

## Review

### Introduction

Polymorphonuclear granulocytes (PMNs; neutrophils) are a type of leukocyte of approximately 10 μm in diameter that play vital roles in the innate immunity response to pathogens. PMNs are the first responders to infection or injury. Persistent neutropenia leads to increased risk of microorganism infections, while excessive recruitment and activation or delayed removal of PMNs results in tissue damage in inflammatory disorders [1]. Following myocardial infarction (MI), numbers of circulating PMNs increase, and the post-MI PMN to lymphocyte ratio has been reported by Akpek and colleagues to predict major adverse cardiac events in MI patients [2]. While PMN counts do not improve the ability to diagnose MI, they are a prognostic biomarker of chronic remodeling of the left ventricle (LV) [3]. Increased PMN counts after percutaneous coronary intervention for ST-elevation MI associates with larger infarct sizes and worse cardiac function [4]. Neutrophil depletion reduces infarct size and the extent of injury in a canine model [5,6]. As such, PMNs have been shown to mediate MI-induced cardiac injury and remodeling. However, the potential mechanisms by which neutrophils regulate MI-induced LV remodeling are not well understood, and PMN depletion strategies in humans increased adverse outcomes post-MI [7]. This review will discuss our current understanding of PMN biology, including recruitment, activation, clearance, and function. We also discuss the roles of PMN-derived components in inflammation and innate immunity, focusing on the MI setting. In addition, we propose future directions that may advance the PMN research arena.

### Biological characteristics of PMNs

PMNs are the most abundant leukocyte cell type in mammals, accounting for ~35-75% of circulating leukocytes under normal conditions [8]. PMNs are the first-line immune cells recruited to sites of injury as a defense against microorganisms. PMN microbicidal mechanisms include receptor-mediated phagocytosis and intracellular killing, release of antimicrobial granule contents by degranulation, and the formation of neutrophil extracellular traps (NETs) [9]. In addition to their antimicrobial activity, growing evidence suggests that PMNs play an essential role in non-infectious inflammation, innate immunity, and tissue remodeling [10].

Based on *ex vivo* evaluation, murine and human PMNs have a circulating lifespan of 5–10 h [11,12]. However, recent work by Pillay and colleagues using *in vivo* PMN labeling has shown that the circulating lifespan of human PMNs can last up to 5.4 days, indicating that *in vivo* characteristics of PMNs may be altered by *ex vivo* manipulation or that *in vivo* stimuli can prevent PMN apoptosis [13]. In the proinflammatory environment, for example, PMN lifespan can be prolonged by tumor necrosis factor (TNF)-α- or interleukin (IL)-1β-stimulated inhibition of apoptosis [14].

PMN development and maturation take place in the bone marrow. In the presence of growth factors and cytokines, pluripotent hematopoietic cells differentiate into myeloblasts, which are the precursor cells of PMNs [15]. PMNs synthesize components stored in different granules as part of the maturation process [10]. It is estimated that PMNs are produced at ~1 × 10^9^ cells per kilogram body weight daily under physiological conditions [16]. Only 1-2% of mature PMNs circulate, while 98-99% remain in the bone marrow [17]. Circulating PMNs are mature, terminally differentiated cells that have lost their proliferative capacity. In response to a challenge, mature PMNs in the bone marrow mobilize into the blood and are recruited to injury sites. PMN chemoattraction is regulated by chemokines, cytokines, and microbial products [1].

### PMN extravasation and recruitment in response to MI

In the setting of MI, chemokines that recruit PMNs to sites of ischemia include macrophage inflammatory protein-2α (MIP-2α, CXCL2, GRO β), leukotriene B4 (LTB4), CINC-1 (CXCL1, GRO α, KC), IL-8 (CXCL8), and complement 5a [18,19]. PMN-attracting CXC chemokines are rapidly and profoundly increased post-MI and have been localized bound to glycosaminoglycans on endothelial cell surfaces or in the extracellular matrix. The accumulation of high concentrations of chemokines at the ischemic site attracts PMNs to the injury area by interaction with cell surface chemokine receptors [20].

PMNs leave the circulation and infiltrate to the infarct region through several sequential steps, collectively known as extravasation. The extravasation of PMNs occurs primarily in post-capillary venules, where hemodynamic shear forces are diminished and the vessel wall is thin. As a first step, PMNs are arrested from the fast-flowing blood stream and roll on endothelial cells. This reaction is mediated through binding of P-selectin ligand 1 and L-selectin constitutively expressed on PMNs to P-selectin, E-selectin, intercellular adhesion molecules (ICAMs), and vascular cell adhesion molecules expressed by activated endothelial cells [15]. Second, firm adhesion occurs by interaction of the β_2_ integrin lymphocyte function-associated antigen-1 (α_L_β_2_, LFA-1, CD11a/CD18) and macrophage-1 antigen (Mac-1, α_M_β_2_, CD11b/CD18, CR3) present on PMNs with their ligands ICAM-1 and ICAM-2 on endothelial cells. Next, PMN transendothelial migration takes place by paracellular or intracellular trafficking. While most PMNs squeeze between endothelial cells (paracellular trafficking), a small fraction penetrates and passes through pores in the cytoplasm of individual endothelial cell (intracellular trafficking) [15]. Mediators that guide migration are the same as those of firm adhesion, namely integrins α_L_β_2_ and α_M_β_2_, ICAM-1, and ICAM-2. PMN homing to the infarct site is similar to PMN extravasation into other tissues as part of a common wound healing response to injury.

**Figure 1:**
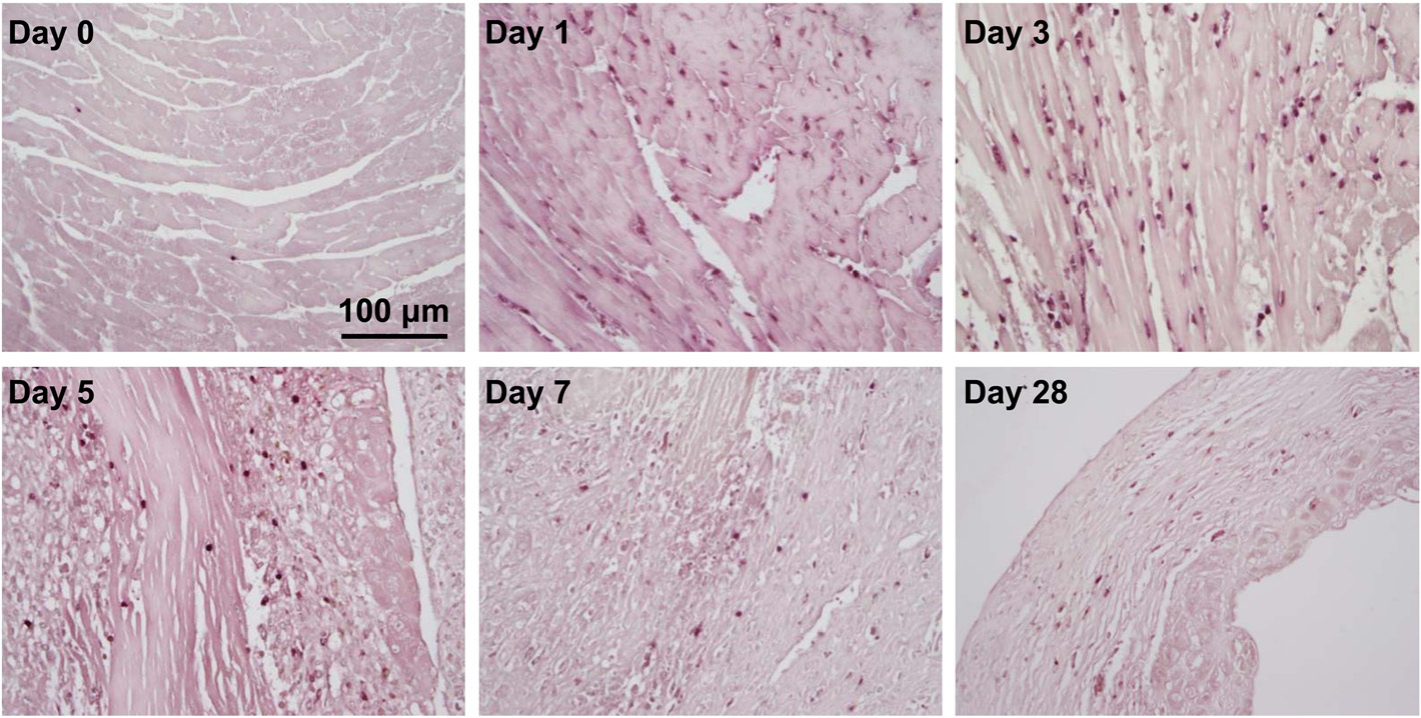
**Time course of PMN infiltration post-MI.** MI was created by permanent ligation of the left anterior descending coronary artery in C57BL/6J mice. Following MI, PMN infiltration peaked at days 1–3, started to decline at day 5, and was present at very low levels from day 7 post-MI. PMNs were stained with anti-mouse neutrophil monoclonal antibody (Cederlane, CL8993AP, 1:100). Representative images from *n* = 3 stained samples per group. Our own unpublished data.

In the absence of reperfusion, PMNs are the first inflammatory cells recruited to the infarct area. With permanent occlusion in C57BL/6J mice, PMN infiltration occurs within hours post-MI, peaks at days 1–3, starts to decline at day 5, and is present at very low levels from day 7 post-MI (Figure 1). As such, PMNs primarily regulate the early LV remodeling response. PMNs initiate the acute inflammatory response to engulf dead cells and tissue debris and facilitate post-MI repair. However, excessive PMN infiltration or delayed regression exacerbates tissue injury by the abundant release of inflammatory mediators and proteinases [21]. Hence, PMN infiltration and removal need to be tightly controlled.

### PMN activation post-MI

In response to infection, PMNs can be activated by pathogen-associated molecular patterns from pathogens or danger-associated molecular patterns (DAMPs) from host tissue via engagement with pattern recognition receptors expressed on the surface or within the cytoplasm of PMNs. PMNs express a wide array of pattern recognition receptors, including 12 of the13 known toll-like receptors (TLRs; the exception is TLR3), C-type lectin receptors dectin 1 (CLEC7A) and CLEC2, NOD-like receptors (NLRs), and cytoplastic sensors of ribonucleic acids, including retinoic acid-inducible gene 1 (RIG-I) and melanoma differentiation-associated protein 5 (MDA5) [22-26]. Activated PMNs kill invading pathogens by the mechanisms of release of reactive oxygen species (ROS) and granule proteins, as well as NETs. However, uncontrolled PMN accumulation can lead to injury to host tissue and cells.

DAMPs are molecules that can initiate and perpetuate the immune response in non-infectious inflammatory conditions, and DAMPs are produced from host tissue or immune cells in response to stress or injury. MI-associated DAMPs include heat shock proteins, high-mobility group box (HMGB)-1, low molecular hyaluronic acid, and fibronectin fragments [27]. DAMPs, as endogenous danger signal and secondary injury-promoting factors, engage with pattern recognition receptors to activate PMNs, other immune cells, or parenchymal cells [28]. This leads to the development of a proinflammatory autocrine loop that can result in chronic or unresolved inflammation. For example, HMGB1, an endogenous ligand for TLR2 and TLR4, is released both actively and passively by injured cells [29]. Injection of HMGB1 results in PMN accumulation, and an anti-HMGB1 blocking antibody inhibits PMN infiltration in lipopolysaccharide-induced lung injury [30]. HMGB1, therefore, promotes and sustains the inflammatory response.

### PMN clearance and resolution of inflammation

Apoptotic PMNs are removed by macrophage- or dendritic cell-mediated phagocytosis. In the absence of infection or inflammation, PMN clearance occurs at significant rates in the spleen, liver, and bone marrow [31]. In response to infection or inflammation, PMNs can infiltrate in and be cleared from all tissues of the body [21]. PMN apoptosis as well as subsequent removal is a hallmark of inflammation resolution, an active process that requires activation of many inhibitory pathway cascades [20]. For example, apoptotic PMNs produce “find me” (e.g., lipid mediators and nucleotides) and “eat me” (e.g., lysophosphatidylcholine) signals to attract scavengers by at least two different mechanisms [20,21]. First, apoptotic PMNs generate annexin A1 and lactoferrin to inhibit PMN infiltration. Moreover, these two mediators attract phagocytic macrophages to remove PMNs. Second, phagocytosis of apoptotic PMNs by macrophages activates an antiinflammatory pathway to inhibit proinflammatory mediators (e.g., TNF-α) and induce the production of IL-10, transforming growth factor-β and pro-resolving lipid mediators such as lipoxins, protectins, and resolvins [32]. These pro-resolving mediators inhibit PMN transendothelial migration and scavenge chemokines and cytokines. Esmann and colleagues have recently shown that after exposure to activating stimuli (e.g., lipopolysaccharide and interferon-γ), PMNs, as a self-regulation mechanism, can ingest apoptotic PMNs and contribute to the resolution of acute inflammation [33]. If not removed in a timely manner, dying PMNs can liberate granule components to the extracellular environment and prolong the ongoing inflammatory response [21]. The importance of these mechanisms in the MI setting, however, needs to be investigated.

### ROS and MI

Upon contact with proinflammatory stimuli (e.g., cytokines and growth factors), PMNs release large amounts of ROS through a process known as the respiratory burst [34]. The respiratory burst is mediated by nicotinamide adenine dinucleotide phosphate (NADPH) oxidase multicomponent enzyme. NAPDH oxidase is composed of a membrane-bound cytochrome b558 consisting of gp91phox and p22phox, cytosolic subunit p67phox, p47phox, and p40phox, and the small G-protein Rac (Rac1 or Rac2) [35]. In resting PMNs, the NAPDH oxidase complex is not assembled. Upon activation, these subunits assemble into an active enzyme complex that catalyzes the production of ROS [34].

The generation of ROS is an indispensable contributor of PMN antimicrobial activity and provides one of the most efficient microbicidal mechanisms [34]. NADPH oxidase increases ROS production. ROS can directly damage host tissue and cells by modifying amino acids, proteins, and lipids to alter their biological functions [10]. For example, ROS can oxidize cysteine residues to regulate the activities of phosphatases, metalloproteinases, and caspases [10]. Antioxidant pre-treatment in rats decreases microvascular density in the infarct region at day 7 post-MI, and inhibition of NADPH oxidase attenuates post-MI cardiac fibrosis in rats or rabbits, indicating pro-angiogenic and pro-fibrotic roles of ROS [36-38]. While an appropriate amount of ROS generation is beneficial to post-MI cardiac repair, excessive ROS are detrimental.

### PMN granule components

**Figure 2:**
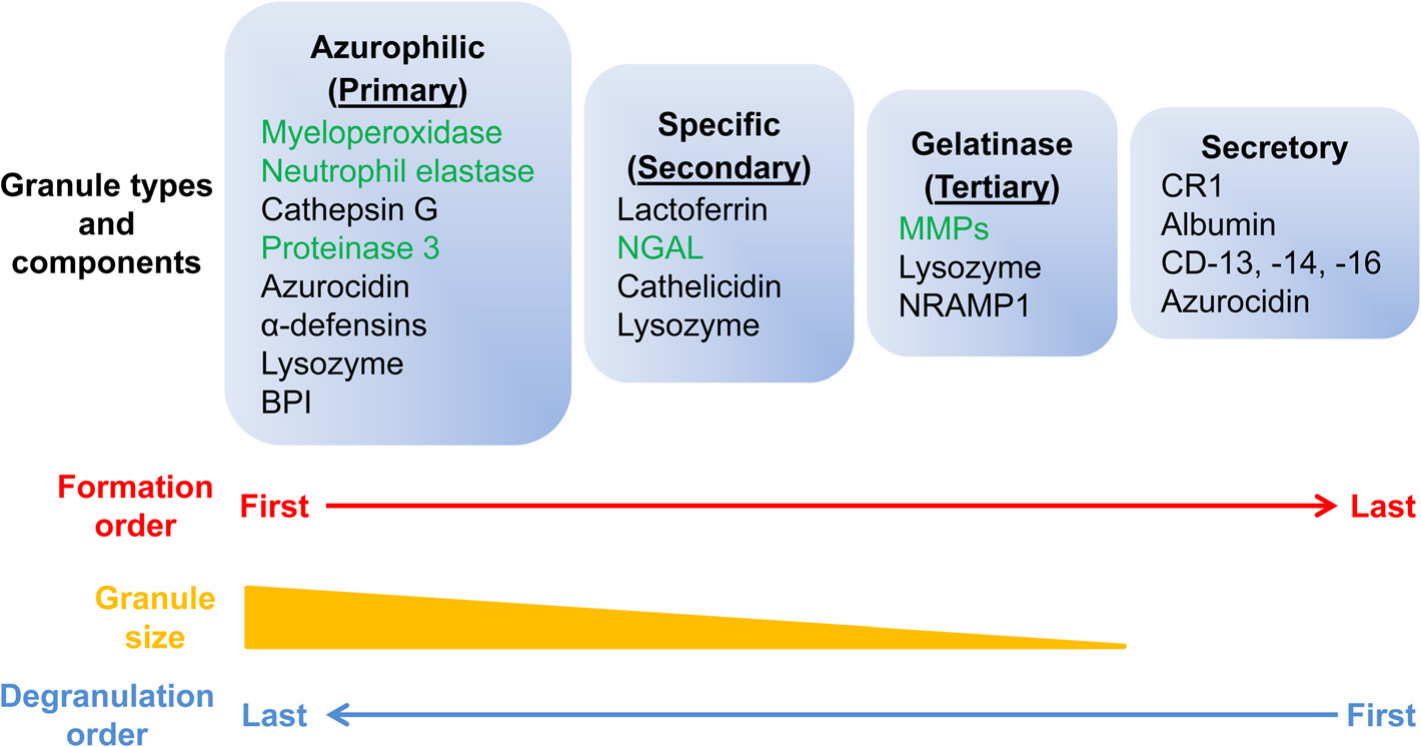
**PMN granules.** The types, components, formation order, granule size, and degranulation order of PMN granules. The granule components that have been evaluated in the MI setting are highlighted in green. BPI: Bactericidal/permeability-increasing protein; NGAL: neutrophil gelatinase-associated lipocalin; NRAMP1: natural resistance associated macrophage protein-1; CR1: complement receptor 1.

PMNs play a critical role in protecting against pathogen infection and non-infectious inflammatory processes, and its functions depend on the exocytosis and release of PMN granule components. There are four types of PMN granules, which combined contain approximately 300 proteins: azurophilic (primary), specific (secondary), gelatinase (tertiary), and secretory granules (Figure 2). Azurophilic granules, the largest, are first formed during PMN maturation and contain myeloperoxidase (MPO), serine proteases, azurocidin, α-defensins, lysozyme, and bactericidal/permeability-increasing protein [10]. Specific granules are smaller than azurophilic granules in diameter and contain lactoferrin, neutrophil gelatinase-associated lipocalin (NGAL, lipocalin-2), cathelicidin, and lysozyme [39]. Gelatinase granules are smaller than specific granules and contain multiple matrix metalloproteinases (MMP-8 and −9 in particular) and a few microbicidal materials. Secretory granules consist primarily of complement receptor 1, plasma protein albumin, CD13 (aminopeptidase N), CD14, and CD16 (Fc gamma receptor III) [10].

PMN granules are sequentially mobilized into the tissue during cell migration. Secretory granules are discharged first, and these components interact with the endothelium and other leukocytes in the circulation. Gelatinase degranulation occurs during transendothelial migration of PMNs, followed by the release of specific and azurophilic granules at the site of inflammation [40]. In addition to antimicrobial functions, these granule components are involved in a number of inflammation-associated diseases, including MI. Below, we summarize the current literature on the roles of granule components in post-MI LV remodeling. For granule components that have not been studied in the MI setting, we discuss their roles in regulating inflammation and innate immunity.

### Granule components evaluated in the MI setting

#### Myeloperoxidase (MPO)

MPO is an enzyme that catalyzes the oxidation of halide ions to hypohalous acids mediated by hydrogen peroxide, which modifies amino acids and many kinds of macromolecules and affects their normal biological properties [41]. In addition to acting as a key component of the oxygen-dependent intracellular microbicidal system, MPO is involved in tissue injury and remodeling. MPO is elevated in MI patients and can act as a diagnostic plasma marker of MI [42]. High MPO is also a risk factor for long-term mortality [43]. Post-MI, MPO is secreted by PMNs and macrophages, and it accumulates in infarct regions to oxidize proteins and lipids. MPO deletion in mice reduces leukocyte infiltration and also attenuates LV function and dilatation, which have been shown in part to be due to decreased oxidative inactivation of plasminogen activator inhibitor 1 [44]. In addition, MPO generates cytotoxic products of glycine (formaldehyde) and threonine (acrolein) in the infarct zone, which adversely affects LV remodeling and function in mice [45]. Reactive chlorinating species produced by MPO catalyze plasmalogens to produce the alpha-chloro fatty aldehyde 2-chlorohexadecanal, which elicits myocardial damage and reduces ventricular performance in rats [46]. Targeting MPO signaling may represent a promising way to alleviate MI-induced LV remodeling.

### Serine proteases

Serine proteases stored in azurophilic granules include neutrophil elastase (NE), cathepsin G, proteinase 3, and neutrophil serine protease-4. Neutrophil serine protease-4 has recently been identified and shows 39% identity to NE and proteinase 3 [47]. In the presence of ROS, serine proteinases can break down internalized pathogens, proteolytically degrade cytokines and chemokines, and activate cell surface receptors [48]. In addition, serine proteinases activate the coagulation cascade and platelets to stimulate thrombus formation [49]. During systemic infection, activation of coagulation facilitates compartmentalization of pathogens in liver microvessels and limits infection expansion. In contrast, in the absence of microorganism challenge, coagulation induces large vessel thrombosis and contributes to a risk for MI and stroke.

NE degrades elastin, collagens, and fibrinogen and contributes to cardiac damage post-MI. NE induces IL-6 secretion to impair cardiac contractility by a nitric oxide-dependent pathway [50]. NE can cleave and activate pro-MMP-9, indicating an interactive action of PMN-derived molecules [51]. NE is released in the early stages of ischemia, and inhibition of NE has been shown to reduce infarct size [52]. Similarly, a selective NE inhibitor protects against myocardial stunning after ischemia/reperfusion in swine [53]. Proteinase 3 is stored in both azurophilic and secretory granules. Proteinase 3 induces endothelial cell apoptosis by caspase-like activity [54], cleaves angiotensinogen to generate angiotensin I and II [55], activates proinflammatory factors (e.g., TNF-α, IL-1β, and IL-18), and degrades extracellular matrix (e.g., fibronectin and collagen IV) [56]. Proteinase 3 levels in the plasma are higher in chronic post-MI patients who later die or are re-admitted for heart failure compared to event-free survivors [56]. This indicates that proteinase 3 may exacerbate heart failure and serve as a prognostic marker.

### NGAL

NGAL is a glycoprotein with bacteriostatic properties stored in specific granules of mature PMNs. In humans, NGAL binds directly with MMP-9 to form a high molecular weight complex, protecting MMP-9 from degradation [57]. This binding occurs at the 87 amino acid of the NGAL, which is a cysteine in humans [58]. Mouse NGAL does not have this cysteine and does not bind directly to MMP-9. NGAL levels significantly increase in both rats and patients post-MI and associate with adverse outcomes [59]. High plasma NGAL before intervention has been shown to independently predict all-cause mortality for MI patients treated with primary percutaneous coronary intervention [60]. The NGAL mechanisms of regulating LV remodeling have not been revealed, but may involve both direct interactions with MMP-9 as well as growth factor functions independent of complex formation.

### MMP-8

Despite originally being classified as the neutrophil collagenase, MMP-8 is secreted not only by PMNs, but also by macrophages [61]. MMP-8 promotes PMN migration by degrading collagens [62], and PMN depletion inhibits early collagen degradation because of the lack of MMP-8 [63]. MMP-8 degrades fibrillar collagen by binding and cleavage of collagen type I α1 and α 2 chains [64]. The quantities of total and active MMP-8 were shown to be higher in patients with LV rupture than those without rupture [65], indicating that MMP-8 may promote infarct rupture in humans by degrading collagen.

### MMP-9

MMP-9 is one of the most widely investigated MMPs in cardiovascular disease. Infiltrating PMNs are an early source of MMP-9 after MI both with and without reperfusion in humans and multiple animal models, including mice, rabbits, and canines [66-69]. PMN-derived MMP-9 is stored in gelatinase granules and released upon chemotactic stimulation. MMP-9 is also be secreted by macrophages, myocytes, fibroblasts, vascular smooth muscle cells, and endothelial cells [61]. MMP-9 is significantly elevated in the first week after MI in mice, consistent with the time course of PMN and macrophage infiltration. MMP-9 deletion attenuates LV dysfunction and collagen deposition and promotes angiogenesis post-MI in mice [70,71]. Neutrophil-derived MMP-9 may exert very early effects in the MI setting by degrading extracellular matrix and promoting leukocyte cell infiltration into the infarct area, while MMP-9 from other cells may regulate scar formation [72,73].

### Granule components that have not been evaluated in the MI setting

#### Cathepsin G

Cathepsin G has biphasic regulation of leukocyte chemotaxis, serving as both a stimulator and repressor of chemotaxis. Substrate availability determines its action, as cathepsin G enhances PMN and monocyte chemotaxis by cleaving the N-terminal residues of CXCL5 and CCL15 to increase their chemotactic activities [74]. Conversely, cathepsin G also degrades CCL5, CCL3, CXCL12, and CXCR4 to reduce PMN and monocyte chemotaxis [75,76]. Cathepsin G is a potent platelet activator and promotes intravascular thrombosis, thus contributing to the formation of a thrombus clot [77].

### Azurocidin

Azurocidin, also known as cationic antimicrobial protein of 37 kDa (CAP37) or heparin-binding protein (HBP), is stored in both azurophilic and secretory granules. Azurocidin is released at both the very early phase and the later phase of PMN recruitment to sites of inflammation [78]. Azurocidin induces monocyte recruitment and enhances cytokine production in monocytes/macrophages, signifying the ability of azurocidin to regulate monocytes/macrophage infiltration and activation in the post-MI setting [79-81]. The effect of azurocidin on leukocytes is dependent on β_2_ integrins and the formyl peptide receptor. Originally considered devoid of proteinase activity, azurocidin can actually cleave insulin-like growth factor binding protein-1, -2, and −4 *in vitro*[82]. The LTB4-induced increase in vascular permeability is mediated by azurocidin [83], suggesting that azurocidin may promote leukocyte extravasation.

### α-defensins

The α-defensins, also referred to as human neutrophil peptides (HNPs), are small cationic antimicrobial peptides mainly present in the azurophilic granules. The α-defensins not only have antimicrobial function, but also possess immunoregulatory properties mediated by direct interaction with innate immune cells [84]. HNP-1 and −2 are potent chemoattractants for monocyte, naïve T cells, and immature dendritic cells, but not for mature dendritic cells or PMNs [85,86]. In addition, HNP-1 is able to activate monocyte-derived dendritic cells and upregulate the production of proinflammatory cytokines [87]. In view of their immunoregulatory activities, future studies to explore the functions of α-defensins in MI are warranted.

### Lactoferrin

Lactoferrin is an iron-binding glycoprotein of the transferrin family present in the specific granules. It is also synthesized by epithelial cells [88]. In addition to direct antimicrobial activity, lactoferrin inhibits the upregulation of adhesion molecules, limits iron-mediated damage to host tissue, suppresses proinflammatory cytokine production, and limits PMN recruitment [89]. Post-MI, lactoferrin may have protective effects by inhibiting excessive inflammation and ROS production.

### Cathelicidin

Cathelicidin, also known as cathelicidin-related antimicrobial peptide (CRAMP) in mouse and LL-37 or hCAP18 in human, resides in specific granules. In addition to potent microbicidal activity, LL-37 inhibits PMN apoptosis and stimulates monocyte recruitment, angiogenesis, and tissue regeneration [90]. LL37 elevates IL-1β-induced release of cytokines (IL-6 and IL-10) and chemokines such as MCP-1, MCP-3, and IL-8 in macrophages [91,92]. LL-37 deposits at sites of endothelial injury, facilitates re-endothelization, and limits neointima formation after stent implantation by enhancing early outgrowth cell recruitment and release of growth factors [93]. Further, stents coated with LL-37 have reduced re-stenosis, indicating that LL-37 may promote the healing response [93]. Doring and colleagues show that lack of CRAMP reduces atherosclerotic lesion size by restraining monocyte recruitment and by reducing the adhesion of classical monocytes and PMNs in a formyl peptide receptor-dependent way [94]. In early stages of atherosclerosis, CRAMP is specifically expressed in PMNs, but not in monocytes or macrophages. Therefore, cathelicidin may modulate LV remodeling after MI by regulating leukocyte infiltration, apoptosis, and angiogenesis.

### MMP-25

MMP-25, also known as MT6-MMP or leukolysin, is a membrane-type MMP. In PMNs, MMP-25 is present in gelatinase granules and is also found in nuclear/endoplasmic reticulum/Golgi fractions [95]. *In vitro* studies show that MMP-25 cleaves CXCL5, CCL15, and CCL23 to activate these chemokines, and thus promotes the recruitment of PMNs and monocytes [96]. MMP-25 roles, however, remain unknown, and MMP-25 levels have not even been measured post-MI.

### NETs

PMNs release granule antimicrobial proteins and nuclear components (DNA, histones) into the extracellular environment that form NETs to trap invading pathogens. This process is referred to as NETosis and is an alternative to PMN apoptosis [97]. NETs degrade virulent factors and kill microorganisms to prevent infection from spreading [98]. NETs also have detrimental influences on the host. NETs activate the complement system, and the complement component C1q can inhibit NETs degradation, thus establishing a positive feedback loop to exacerbate disease progression [99]. It has been shown that NETs facilitate thrombosis in MI patients, probably by promoting fibrin deposition and platelet aggregation [100]. The role of NETs in the progression of MI-induced heart failure, however, has not been investigated.

## Conclusions

**Figure 3:**
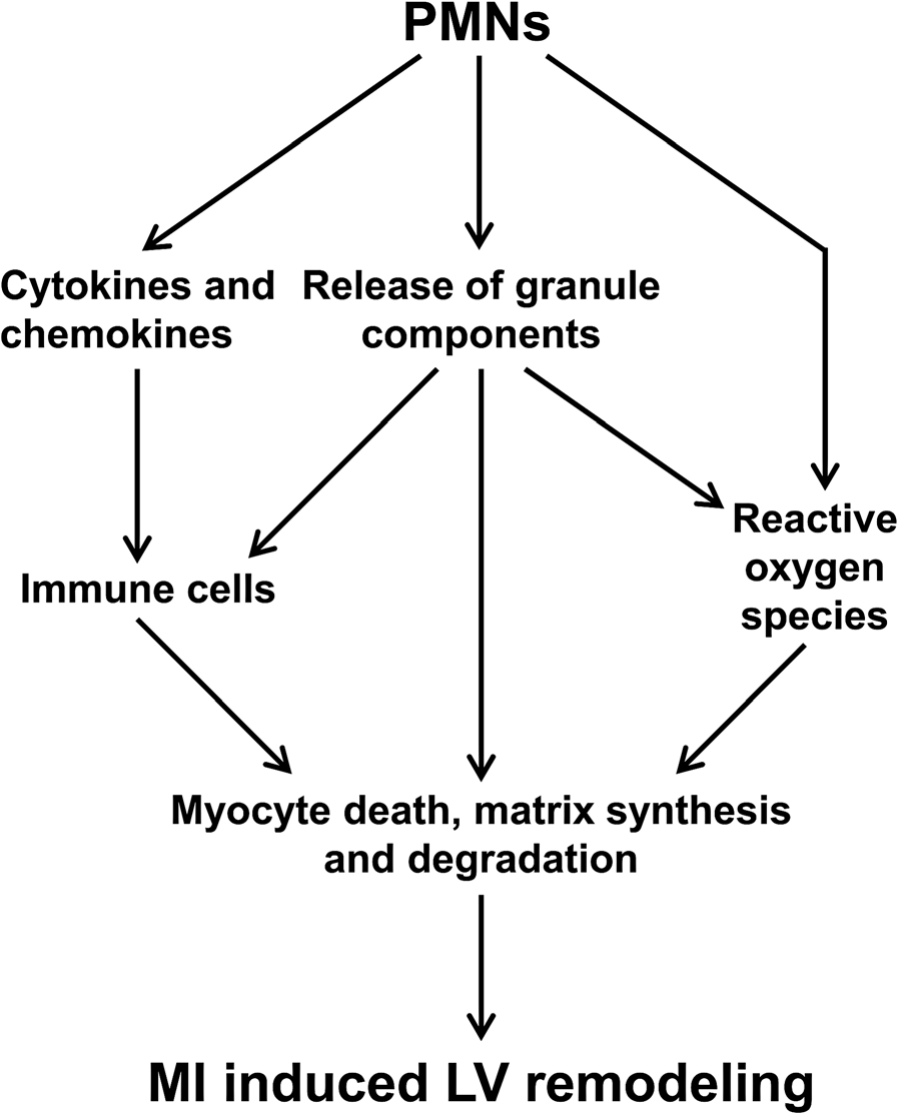
**Mechanisms of action of PMNs on post-MI LV remodeling.** Infiltrating PMNs release a wide range of cytokines and chemokines, granule components, and reactive oxygen species, which directly and indirectly regulate immune cell infiltration and function to modulate remodeling response.

This review summarizes the roles of PMNs and PMN-derived granule components in inflammation, innate immunity, and MI. PMNs regulate the post-MI wound healing response through several mechanisms (Figure 3). PMNs are activated by cytokines and chemokines, and activated PMNs in turn release cytokines and chemokines to potentiate the inflammatory component of wound healing [101]. PMN degranulation releases an array of proteases that regulate LV remodeling by modulating immune cell infiltration and function, including ROS production. The PMN respiratory burst generates ROS to directly modify biological molecules. However, several aspects remain to be elucidated in order to better understand PMN roles after MI.

First, PMN roles post-MI need to be better understood, using systematic approaches that distinguish the negative and positive roles. In order for therapeutic strategies to be developed that promote healing while preventing adverse remodeling, we need to better understand the complexity of PMNs in mediating the early inflammatory response.

Second, there may be different activation phenotypes of PMNs following MI [102-104]. A recent study by Fridlender and colleagues suggests that tumor associated PMNs can be polarized towards different phenotypes [104]. Blocking TGF-β slows tumor growth by increasing the influx of PMNs to produce higher levels of proinflammatory cytokines, which are more cytotoxic [104]. PMN depletion without TGF-β blockade, however, also decreases tumor growth. TGF-β, therefore, promotes a PMN pro-tumor phenotype, while blocking TGF-β induces a PMN anti-tumor phenotype [104]. TGF-β effects on tumors and the post-MI LV are likely opposite, as TGF-β promotes post-MI infarct healing and blocking TGF-β increases MI-induced mortality and LV dilation [105]. PMN phenotypes should be examined by isolating PMNs from post-MI hearts at different time points and measuring the expression of key effector molecules. Before this can be accomplished, however, we need to determine what markers can be used to differentiate phenotypes and whether overall inflammatory status is sufficient.

Third, whether PMNs directly or indirectly regulate macrophage polarization (M1 or M2 activation) or function is not currently well understood. This could be evaluated by incubating resting macrophages with conditioned media from activated PMNs and monitoring the macrophages for M1 and M2 markers [106]. It may be that PMNs from different post-MI times promote differential macrophage activation patterns.

Fourth, whether PMNs regulate cardiac fibroblast phenotype and post-MI scar formation is not known [107]. The role of macrophages in activating fibroblasts has been studied, but whether PMNs exert similar or different activation functions is unknown. This can be addressed by incubating isolated cardiac fibroblasts with activated PMNs and measuring fibroblast phenotype and secretion of extracellular matrix [106].

In conclusion, understanding how PMNs regulate post-MI LV remodeling may provide promising intervention targets for MI patients. Understanding the detrimental and beneficial roles will provide mechanistic insight into how PMNs regulate inflammatory responses, both in the MI setting and in other diseases that have inflammation as a common response.

## Abbreviations

DAMPs: Damage-associated molecular patterns; HNPs: Human neutrophil peptides; ICAMs: Intercellular adhesion molecules; IL: Interleukin; LTB4: Leukotriene B4; LV: Left ventricle; MCP-1: Monocyte chemoattractant protein-1; MI: Myocardial infarction; MMPs: Matrix metalloproteinases; MPO: Myeloperoxidase; NE: Neutrophil elastase; NETs: Neutrophil extracellular traps; NGAL: Neutrophil gelatinase-associated lipocalin; ROS: Reactive oxygen species; TLR: Toll-like receptor; TNF: Tumor necrosis factor.

## Competing interests

The authors declare that they have no competing interests.

## Authors’ contributions

YM and MLL conceived the concept of the review. YM wrote the first draft. YM, AY, and MLL edited and revised the manuscript. All authors read and approved the final manuscript.
